# Health and Behavioral Survey of over 8000 Finnish Cats

**DOI:** 10.3389/fvets.2016.00070

**Published:** 2016-08-29

**Authors:** Katariina Vapalahti, Anna-Maija Virtala, Tara A. Joensuu, Katriina Tiira, Jaana Tähtinen, Hannes Lohi

**Affiliations:** ^1^Department of Veterinary Biosciences, University of Helsinki, Helsinki, Finland; ^2^Research Programs Unit, Molecular Neurology, University of Helsinki, Helsinki, Finland; ^3^The Folkhälsan Institute of Genetics, Helsinki, Finland; ^4^Health and Education Committee of Finnish Cat Association, Helsinki, Finland

**Keywords:** health survey, feline, epidemiology, disease, behavior, questionnaire

## Abstract

A comprehensive feline health survey was conducted to reveal breed-specific inheritable diseases in Finnish pedigree cats for genetic research. Prevalence of 19 disease categories and 227 feline diseases were defined in a study population of 8175 cats belonging to 30 breeds. Dental and oral diseases, with a prevalence of 28%, and dental calculus and gingivitis (21 and 8%, respectively) were the most prevalent disease category and diseases among all cats and in most of the breeds. An exception was Korats, which were more often affected by the diseases of the respiratory tract (23%) and asthma (19%). Other prevalent disease categories affected various organ systems, such as the skin (12%), the urinary system (12%), the digestive tract (11%), eyes (10%), the musculoskeletal system (10%), and genitals of female cats (17%). Prevalent health or developmental issues included repetitive vomiting (4%), tail kink (4%), feline odontoclastic resorption lesion (4%), urinary tract infections (4%), as well as cesarean section (6%) and stillborn kittens (6%) among female cats. We found 57 breed-specific conditions by Fisher’s exact tests and logistic regression analyses, including 32 previously described and 19 new breed-specific diseases. The genetic defect has already been found in six of them: polycystic kidney disease, progressive retinal atrophy, hypertrophic cardiomyopathy, and three types of tail malformations. Behavioral profiling revealed breed-specific traits, such as an increased human avoidance in British Short and Longhairs and a higher level of aggression in Turkish vans. Our epidemiological study reveals the overall health profile in Finnish pure and mixed breed cats and identifies many breed-specific conditions without molecular identity for genetic research.

## Introduction

The annotation of the cat genome, in 2007, has facilitated genetic research with novel genomic resources (1, 2). Pedigree cats form appropriate populations for genetic studies, since each breed represent a group of genetically similar animals that descended from few ancestors (3). It has been estimated that 85% of all current breeds have arisen only in the past 75 years, largely due to intentional breeding to influence esthetic qualities (4). There are over 40 officially approved cat breeds according to The Cat Fanciers’ Association^1^ (CFA), whereas The International Cat Association^2^ (TICA) accepts about 60, and the Finnish Cat Association^3^ (Suomen Kissaliittory) – 44 registered breeds.

The cat is the most common pet in Europe, with an estimated 100 million domesticated cats (5). In Finland, there is a cat in every seventh household, comprising almost 600,000 cats in 360,000 households (6). The non-pedigree domestic cat forms the most common group, while Ragdolls lead the pedigree group, according to the Finnish Cat Association registry.

Over 300 genetic diseases have been described in cats, of which ~70% are potential models for human diseases (7). Currently, over 55 genes have been found in various traits (7). A commercial DNA test is available for >10 inherited feline diseases (2). Examples of these are the test for polycystic kidney disease (PKD1) (8), which is common in Persian and Exotic breeds, and the tests for hypertrophic cardiomyopathy (HCM) (9, 10). In addition, over 20 mutations associated with uncommon feline diseases have been localized (2). However, the molecular background of many conditions remains unsolved.

A growing biobank of cats with over 4000 samples has been established in our laboratory at the University of Helsinki to facilitate feline genetic research and to identify genetic causes of breed-specific inherited diseases and behavioral traits. Toward this aim, a comprehensive owner-completed health survey was developed to explore the overall health profiles in pedigree and non-pedigree cats. Besides general information of the cat and their living environments, the health survey comprises questionnaire data, covering altogether 227 feline diseases as well as data on behavioral traits, vaccination, clinical examinations, and possible gene tests.

The present study analyzes the survey-based health profiles in 8175 Finnish cats to identify common and breed-specific conditions. Traits that are enriched in a particular breed or phylogenetic group suggest genetic susceptibility and could be prioritized for sample recruitment. The comprehensive health profiling provides useful data not only for genetic research but also for feline breeding programs and veterinary epidemiology. The survey data inform key health issues in different breeds to guide future research focus and feline biobanking activities.

## Materials and Methods

### Study Design and Questionnaire

A cross-sectional online feline health survey (Presentation S1 in Supplementary Material) was established to collect information about Finnish cats. Besides data on various disorders, breed-specific behavior, living habits (including diet, outdoor activity, contacts with other animals, and hunting), and reproductive fitness were collected in the questionnaire. The survey was targeted at all Finnish cats, including pedigree and non-pedigree domestic cats. Owners were allowed to also report deceased cats.

The cat breeders and owners were informed about the survey in several ways, including advertisements by the Finnish Cat breed Association, social media such as Facebook, and by sharing information bulletins in different cat shows and meetings of the breed clubs. Numbers with the set breed target numbers of submitted responses were published regularly in the research group’s website^4^ and Facebook site to encourage participation.

The minimum sample size required for each breed to estimate disease prevalence was calculated using Epitools calculator (11) and taking into account the annual registration numbers over the past 10 years (2001–2011) by the Finnish Cat Association. To maximize the required sample size for significance, we assumed 50% disease prevalence, which is the most difficult to detect (12). Closely related breeds were grouped together, if there were not enough cats within a breed to reach the minimum sample size requirement.

The questionnaire was divided into 25 sections, which covered basic information about the cat, its owner, possible offspring, living environment, feeding manners, personality and personal habits, and possible genetic and clinical tests, such as ultrasound, vaccination records, and health status, with respect to each disease category. The sections used in this study are described in more detail. The basic information of the cat included the breed, registration number (registered in the Finnish Cat Association, TICA, or CFA), date of birth, and possible death, gender, and birth control status (castration, hormone implant, or contraceptive pill combined). There were 19 disease categories (1) behavioral traits, (2) congenital developmental disorders, (3) dermatological/glandular diseases, (4) ocular diseases, (5) otic diseases, (6) dental and oral diseases, (7) diseases of the urinary system, (8) disorders of the cardiac and circulatory system, (9) blood disorders, (10) diseases of the musculoskeletal system, (11) diseases of the digestive tract, (12) diseases of the respiratory tract, (13) diseases of the nervous system, (14) genital diseases, (15) endocrine and metabolic diseases, (16) autoimmune diseases, (17) tumors (including benign tumors and cancers), (18) parasites and protozoans, and (19) diseases not mentioned in the previous categories. In all these categories, the participant was asked to report whether a given diagnosis was (1) made by a veterinarian, (2) made by the owner, (3) no such diagnosis was made, or (4) this was unknown. There were altogether 227 different diagnoses in the disease categories, varying from 3 to 27 per category.

The questions concerning the cat’s personality covered general activity, contact with people, aggressiveness toward strangers, family members, or other cats, sensitivity toward new situations or unfamiliar people, and possible compulsions, such as licking.

Multiple and incomplete responses were removed from the survey data. If the cat had been reported to belong to a certain breed, but the registration number was not given, the registration number was requested from the owner, the Finnish Cat Association, or searched on the internet. If the registration number was not available, the pedigree cat was moved into a special group, “Others,” as a separation from the verified pedigree and non-pedigree cats. The same procedure was used to investigate the dates of birth, which were missing or apparently wrong (the given date of birth was after the date of response or after date of death). If the date could not be verified, it was left missing.

The ages of the living cats were counted as a difference between the date of response and the date of birth converted to years. For deceased cats, the age of death was similarly counted as a difference between the date of death and the date of birth. Five age groups were created: (1) <1 year, (2) 1 to <3 years, (3) 3 to <7 years, (4) 7 to <11 years, and (5) ≥11 years. The variable “Age” was determined as either the age of the living or the age of the cat at death. Also, a new variable “Alive/dead,” representing whether the cat was alive or dead, was created. Distributions of age, sex, alive/dead cats, and neutered/non-neutered cats were computed for all cats and in each breed. Before commissioning the final health survey, a pilot study was performed on Norwegian Forest cats (*N* = 604) (13) to test the content, usability, and advertisement strategies of the questionnaire.

### Prevalence Estimates

Prevalence with 95% confidence intervals were used to express: (a) the number of cats having had disease/diseases belonging to a disease category (*n* = 19), and (b) the number of cats having had a separate disease (*n* = 227). In addition to the entire study population, prevalence were also determined for each breed and for subgroups of age, sex, neutered/non-neutered cats, and alive/dead cats. Prevalence of genital diseases were counted in subgroups of female or male cats depending on the disease. Phylogenetic grouping was used in tabulating disease prevalence (4, 14). In the prevalence tables, coloring (green = low, yellow = moderate and red = high prevalence) was used to point out the differences between breeds and categories. Prevalence in *disease categories* were counted in three ways: (1) combining diagnoses by veterinarian and owner, (2) diagnoses by veterinarian solely and (3) diagnoses by owner solely. Prevalence in *separate diseases* were also tabulated by breed to find the most common diseases in the breed.

### Breed Specificity

The breed-specific diseases were tentatively deducted from the prevalence of the diseases. The disease was considered breed-specific, if the prevalence of the disease (according to coloring mentioned above and prevalence remainder) distinctly differed (upwards) in one or a few breeds from other breeds among all cats and in subgroups of sex, age, alive/dead cats, and neutered/non-neutered cats. If the disease was found to be breed-specific, according to prevalence, it was tested using the Fisher’s exact test to verify the breed specificity compared with other pedigree cats (combined) and non-pedigree cats. The *p*-values < 0.05 of the Fisher’s exact test were considered to indicate statistically significant difference, and, thus, potential breed specificity of the disease. No correction for the error rate α for false significance in multiple testing (15) was performed, as breed specificity analyses were aimed for preliminary information, which were later controlled in a logistic regression analysis.

In addition, for each tentative breed-specific disease, a logistic regression analysis with random intercept variable “Breed” was performed to observe the effect of the breed in acquiring the disease (Model 1). The model also included variables “Age” (reference group “3 to <7 years”), “Sex,” and “Alive/dead” as fixed (level 1) independent explanatory variables to control the effect of these variables. The effect of “Breed” was measured by the median odds ratio (MOR) (16), which was counted from variance δ^2^ of the random variable “Breed.” MOR expressed (in this study) the MOR between a cat of a higher prevalence and a cat with a lower prevalence, when the cats with the same covariates were randomly chosen from two different breeds.

A logistic regression analysis was also performed without the random variable “Breed” (Model 2) in order to compare the goodness of fit between models 1 and 2. An evidence ratio (17) was used in the comparison. Furthermore, logistic regression analysis was performed with “Breed” included as a fixed variable (Model 3). In this model, each breed, which was suspected to be overrepresented compared with other breeds (as determined by the intercept of the breed in Model 2), formed a separate category. Non-pedigree cats formed their own category, and the rest of the breeds were combined to one category and set to a reference group. The purpose of Model 3 was to display the effects of the overrepresented breeds in acquiring the disease by odds ratios (OR). OR indicated how much higher the odds were in the breed/breeds that were suspected to be prone to disease than in the other breeds.

The models were basically built from the entire study population included, but, to assess the effect of the non-pedigree cats in heterogeneity of breeds (MOR), they were also built from a population of which non-pedigree cats were excluded. In some diseases, separate age groups or non-pedigree cats had to be excluded because of too few or no cases in the group. In such cases, the exclusion was executed in all three models to retain the goodness of fit comparability with Akaike Information criterion (AIC), which requires equal data sets (17). In genital diseases, the models were built from subgroups of female or male cats, and, for female cats, non-pedigree cats were excluded. The models were tested for interactions. If an interaction was detected, the MOR, evidence ratio, and OR of overrepresented breeds yielded from Models 1–3 with the interaction added were compared with corresponding values yielded from models without interactions included, to find out whether the interaction had any effect on these measures.

Median odds ratio and evidence ratios were used to evaluate the breed specificity of the disease. MOR always has values greater or equal to one. The closer to one the MOR was, the less heterogeneity there was between the breeds. The evidence ratio, in turn, indicated how many times more likely Model 1 was to be the best model as compared with Model 2, according to AIC values (18). Since the smaller value of AIC indicated better goodness of fit in terms of Kullback–Leibler discrepancy (19), the evidence ratio was counted only in cases when the AIC value of Model 1 was smaller. Otherwise, Model 2 was considered the better model, and the breed was interpreted not to improve the model.

Neither MOR nor the evidence ratio has any distinct cut point (threshold), which would designate that variable “Breed” had had a significant effect in acquiring the modeled disease (16, 17). In case of low value of the evidence ratio (<10) and a high value of MOR, the deduction of breed specificity was based on the whole entity of the data concerning the disease. In this deduction, in addition to MOR and the evidence ratio, the number of diseased cats, the distribution of the disease among all breeds, and the effect of non-pedigree cats in the model were used. According to the results of the logistic regression analysis and previous studies, the suspected breed-specific disease were divided into four groups: diseases with (1) established heredity, (2) known breed-specific conditions, (3) breed specificity suspected by our study for the first time, and (4) breed specificity not verified in our study.

In Turkish Vans and breed groups of Cymric–Manx and Persian–Exotic, there were several cats of which the date of death was before 2005 (indicating a low possibility of these cats having lived at the same time as the alive cats of our study). To eliminate potential bias caused by deceased cats far in the past, the main results of logistic regression analyses concerning these breeds were verified in a subgroup in which cats died before January 1st, 2005 were excluded as well. Furthermore, in Cornish Rexes, Korats, Sphynxes, Bengals, Turkish Vans, and Europeans, more than 15% of the cats were associated with the same breeder. In potential breed-specific diseases, the prevalence were also counted excluding these breeders to check the effect of the breeder.

### Behavioral Traits

Owners were asked to rate behavior at five levels in the questionnaire (“not at all,” “a little,” “quite a bit,” “often,” and “very often”), and a binary variable was created using, in case of variables concerning aggressiveness and licking, combined levels “often” and “very often,” and in case of variables concerning sensitivity, level “often” to form the first category, while the rest of the levels together formed the reference category. In the case of variables “activity” and “takes contact with people,” combined levels “not at all” and “a little” were used as the extreme features, forming the first category. Prevalence with 95% confidence intervals were computed and tabulated in a similar way to the diseases and Fisher’s exact tests and logistic regression were used in the analysis to verify behavioral differences between breeds.

### Statistical Analyses

All statistical analyses were performed by SAS version 9.3, SAS Institute, Cary, NC, USA. Proc Freq statement with chisq and fisher options was used in testing differences and association in categorical data. Proc Npar1way statement with Wilcoxon option (producing Kruskal–Wallis test) was used in (not normally distributed) continuous data. Proc Logistic and Proc Glimmix statements were used in logistic regression analysis in testing breed specificity of the diseases.

## Results

### Demographics of the Survey Data

**Figure 1:**
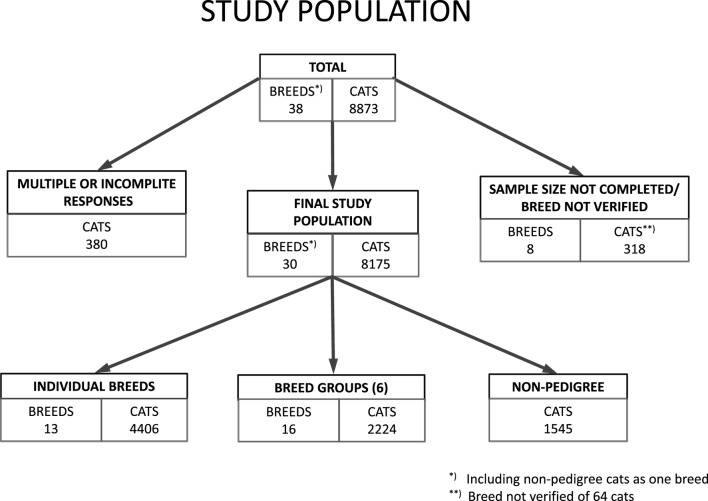
**Population flow chart: cats selected into the study from responses submitted into the feline health study**.

A total of 8873 survey responses were collected, of which 8175 responses were accepted for statistical analyses after quality control (Figure 1). The data were accumulated from 29 different breeds and non-pedigree cats. When the small phylogenetically related breeds with few cats were grouped together, the study included 13 breeds, 6 breed groups (16 breeds), and non-pedigree cats. The largest populations were non-pedigree cats (*N* = 1545), Abyssinian–Ocicat–Somali group (*N* = 539), Siamese–Balinese–Oriental–Seychellois group (*N* = 468), and the Cornish Rex breed (*N* = 437). The sample size for the Cymric–Manx breed remained incomplete with 15 missing responses from the set breed target, and the results should, therefore, be considered only directional in this breed (Table 1).

**Table 1:** **Summary of the breed-specific sample size requirements and response numbers in the health survey**.

Breeds and breed groups	Responses	Minimum sample size	Extra/missing
**Breeds included in the study**			
Abyssinian–Ocicat–Somali	539^a^	329	210
Siamese–Balinese–Oriental–Seychellois	468^b^	358	110
Cornish Rex	437	330	107
Turkish Van	233	157	76
British	378	323	55
Maine Coon	405	350	55
Norwegian Forest cat	406	351	55
Ragdoll	418	365	53
Russian Blue	342	295	47
Bengal	317	272	45
Birman	396	354	42
Siberian–Neva Masquerade	344^c^	310	34
Sphynx	272	238	34
Persian–Exotic	378^d^	348	30
Burmese–Burmilla–Singapura	350^e^	323	27
Korat	242	223	19
European	267	260	7
Devon Rex	293	290	3
Cymric–Manx	145^f^	160	−15
Non-pedigree cat	1545	–^i^	
All	8175		
**Breeds not included in the study**			
American shorthair	2	–^i^	
Sokoke	0	12	−12
German Rex	0	15	−15
Snowshoe	0	15	−15
Selkirk Rex	8	34	−26
Kurilian Bobtail Short- and Longhair	15^g^	57	−42
Egyptian Mau	16	88	−72
Don Sfinx–Peterbald	17	91	−74
Chartreux	28	108	−80
American Curl Short- and Longhair	119^h^	200	−81
Turkish angora	49	171	−122
Others	64	–^i^	
All	318		
**All cats**	8493		

*Minimal required sample size was estimated based on the 10-year registration numbers and 50% disease prevalence*.

*^a^Abyssian (*N* = 179), Ocicat (*N* = 226), Somali (*N* = 134)*.

*^b^Siamese (*N* = 139), Balinese (*N* = 57), Oriental Shorthair (*N* = 227), Oriental Longhair (*N* = 31), Seychellios Shorthair (*N* = 12), Seychellios Longhair (*N* = 2)*.

*^c^Siberian (*N* = 332), Neva Masquerade (*N* = 12)*.

*^d^Persian (*N* = 334), Exotic (*N* = 44)*.

*^e^Burmese (*N* = 303), Burmilla (*N* = 47)*.

*^f^Cymric (*N* = 41), Manx (*N* = 104)*.

*^g^Kurilian Bobtail Shorthair (*N* = 1), Kurilian Bobtail Longhair (*N* = 14)*.

*^h^American Curl Shorthair (*N* = 37), American Curl Longhair (*N* = 82)*.

*^i^No sample size defined*.

The overall study population included more female (53%) than male cats (47%) (chi square *p* < 0.0001) (Data Sheet S1 in Supplementary Material). Also, there were more neutered (72%) than non-neutered cats (27%), and more alive (86%) than dead cats (12%) (*p* < 0.0001 between both subgroups). The mean age of the cats was 5.4 years. The ages of alive and dead cats (mean 4.9 and 9.3 years, respectively), female and male cats (mean 5.6 and 5.2 years, respectively), and neutered and non-neutered cats (mean 6.5 and 2.4 years, respectively) differed significantly (*p* < 0.0001 of Kruskal–Wallis test between each group). The proportions of alive and dead cats did not differ significantly in subgroups of male and female cats, but significantly more in male cats (78%) than female cats (67%), and living cats (77%) than dead cats (71%) were neutered (*p* < 0.001 between both subgroups) (data not shown).

**Figure 2:**
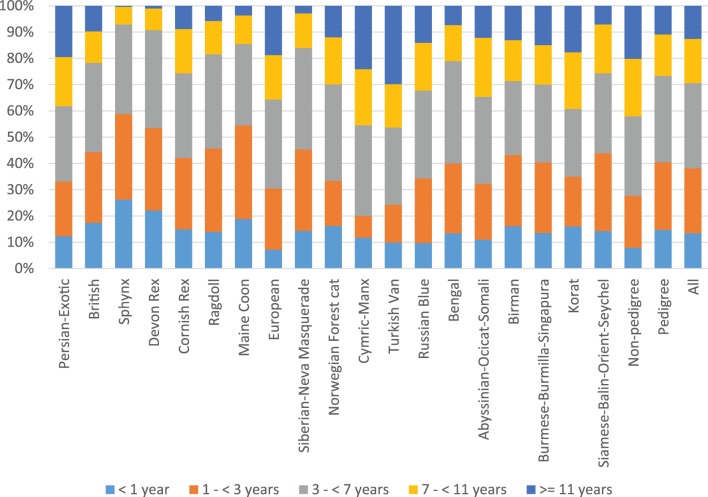
**Age distributions of all cats in all breeds**.

There was significant variation in the age distribution in different breeds (*p* < 0.0001 in both Kruskal–Wallis test for continuous ages and chi square test for age groups) (Figure 2 and Data Sheet S1 in Supplementary Material). For example, 30% of the Turkish vans, but less than 1% of the Sphynxes, belonged to the oldest age group of ≥11 years. The distributions of sex, alive/dead, and neutered/non-neutered cats varied considerably between breeds (*p* < 0.0001 for each). However, there was no significant difference in sex distributions (*p* = 0.1024), when Europeans and breed group of Burmese–Burmilla–Singapura were excluded, indicating that most of the variation originated from these two breeds. The largest proportion of deceased cats was among Turkish vans (28%), and the smallest – in the Siberians–Neva Masquerades group (2%). The highest proportion of neutered cats was found in non-pedigree cats (92%), and the lowest in the Persian–Exotic group (54%).

There were altogether 227 responses, of which either the age or the sex (or both) of the cat was missing. The neutering status was missing from 51 cats. In disease categories, in all other categories, 3–9 were missing values, except in “Other diseases,” where the number was 20. When the missing values of sex, age, and disease category (variables used in logistic regression models) were summed, the sample size requirements were still accomplished for all other breeds, except Cymric–Manxes. The same was true when excluding cats that had died prior to January 1st, 2005 (*n* = 146).

In the behavioral part, information concerning “activity” was missing from 112, “contact with people” from 167, “aggressiveness toward family” from 108, “aggressiveness toward strange people” from 119, “aggressiveness toward other cats” from 207, “sensitivity toward new things” from 115, “sensitivity toward new people” from 113, and “licking” from 154 cats. The effect of missing values on the final results was not investigated, as the behavioral characterization was only preliminary and needs a more comprehensive study in the future.

### Prevalence of Disease Categories and Separate Diseases

#### Prevalence of Disease Categories

**Figure 3:**
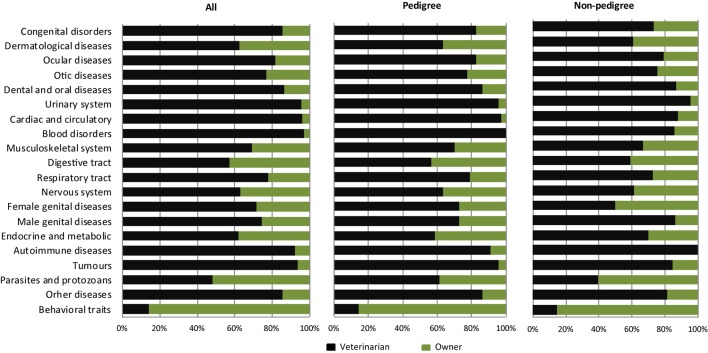
**Proportions of disease categories diagnosed by the veterinarian or the owner**.

Altogether, 5415 cats (66%) were reported to have had a disease/diseases belonging to at least one of the 19 disease categories, including behavioral traits. The disease categories included diagnoses made either by the veterinarians or the owners (see Questionnaire in Presentation S1 in Supplementary Material). Diagnoses in the categories of “behavioral problems” and “parasites and protozoan” had mainly been made by the owner, while the diagnoses in all other disease categories had been mainly made by veterinarians (Figure 3). In the categories of “diseases of the urinary system,” “disorders of the cardiac and circulatory system,” “blood disorders,” “autoimmune diseases,” and “tumors,” almost all of the diagnoses had been made by the veterinarian.

A total of 63% of pedigree cats had been diagnosed with a disease/diseases in at least one of the categories (diagnosed either by the veterinarian or the owner), whereas the percentage among non-pedigree cats was 78%. Among male and female cats, the percentages were 67 and 66%, respectively. The difference between pedigree and non-pedigree cats was significant (chi square *p* < 0.0001). However, when only veterinary diagnoses were considered, and diagnoses in the category of “parasites and protozoans” were excluded, the difference was non-significant (*p* = 0.0745), despite a modest overrepresentation of the non-pedigree cats (55%) compared with pedigree cats (52%). The difference between male and female cats was not significant (*p* = 0.3706); however, when diagnoses in the category of “Genital diseases” were excluded, significantly higher prevalence was found in male (66%) than female cats (59%) (*p* < 0.0001).

The main prevalence results in disease categories diagnosed either by veterinarian or owner are presented below, and in the Data Sheet S2 in Supplementary Material, separately for diagnoses made only by veterinarian or owner (as well as for other subgroups).

**Figure 4:**
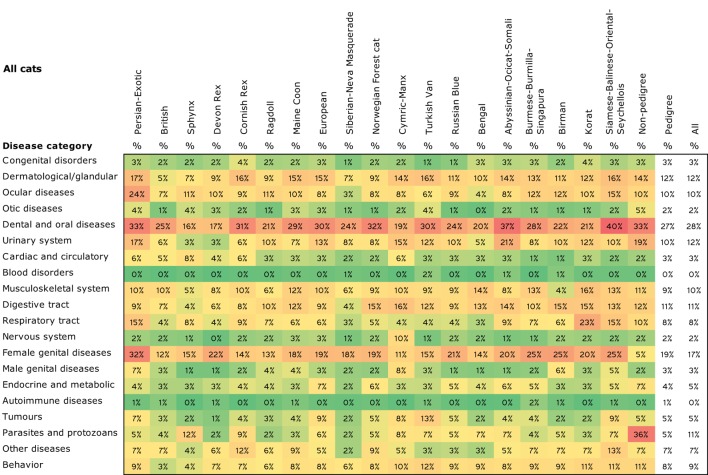
**Prevalence of disease categories in all breeds and breed groups**. Prevalence are colored with gradient filling from the lowest value to the highest (low = green, high = red) to point out the differences between the breeds. Phylogenetic grouping has been used in ordering the breeds.

The most prevalent disease category among all cats was “Dental and oral diseases” (28%, Figure 4). It was the most common category in breeds and subgroups as well (Data Sheet S2 in Supplementary Material). Other prevalent categories included skin disorders (12%), the urinary system (12%), the digestive tract (11%), eyes, (10%), and the musculoskeletal system (10%). Genital diseases were also common in female pedigree cats (19%), but not as frequent in non-pedigree cats (5%) (chi square *p* < 0.0001).

##### Age

Prevalence increased by age in many disease categories. This was particularly apparent in the category of “dental and oral diseases,” but also distinctively in the urinary tract, the digestive tract, the skin, and tumors categories. In contrast, the prevalence of “congenital developmental disorders,” “blood disorders,” and “autoimmune diseases” was not affected by age, while some decrease was observed in the category of “other diseases.” The difference in the prevalence between the age groups of <1 year and ≥11 years was significant in all disease categories, except in “congenital developmental disorders” and “male genital diseases.”

##### Sex, Alive/Dead, and Neutering

In almost all the disease categories, male cats were overrepresented, as compared to female cats, deceased as compared to living cats, and neutered as compared to non-neutered cats. Between male and female, the differences were significant in the categories of “dental and oral diseases,” “genital diseases,” “diseases of the digestive tract,” “diseases of the urinary system,” “diseases of the respiratory tract,” “tumors,” “parasites and protozoans,” and “cardiac and circulatory system.” Of these categories, female cats were overrepresented only in the categories of “genital diseases” and “tumors.” Between alive and deceased cats, the differences were significant in all other categories, except in “female and male genital diseases,” “behavioral problems,” and “parasites and protozoans,” and between neutered and non-neutered cats, in all other categories, except in “other diseases” and “blood disorders.” In categories with significant difference, deceased cats had higher prevalence than living cats, and neutered cats – higher than non-neutered cats.

##### Pedigree and Non-Pedigree

**Figure 5:**
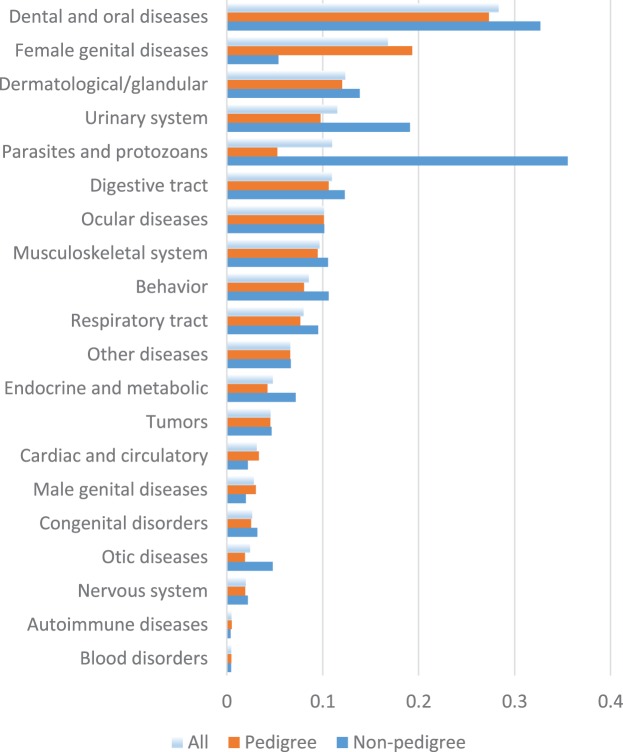
**Bar chart of prevalence of disease categories among all cats, pedigree cats, and non-pedigree cats**.

Non-pedigree cats were overrepresented as compared to pedigree cats in most of the disease categories (Figure 5). They had significantly higher prevalence in “dermatological/glandular diseases,” “diseases of the digestive tract,” “diseases of the urinary system,” “behavioral problems,” “diseases of the respiratory tract,” “parasites and protozoans,” “otic diseases,” and “dental and oral diseases” as compared to pedigree cats. Pedigree cats had significantly higher prevalence only in the “genital diseases” and “disorders of the cardiac and circulatory system” categories.

##### Individual Breeds

In separate breeds, like stated above, the most prevalent disease category in most breeds was “dental and oral diseases” with a minimum prevalence of 16% in Sphynxes and maximum – 40% in the breed group of Siamese–Balinese–Oriental–Somali. Non-pedigree cats had the highest prevalence in the category of “parasites and protozoans” (36%) (Figure 4).

The most prominent breed-specific exceptions included high prevalence in “ocular diseases” (24%) and “female genital diseases” (32%) in Persian–Exotics, “respiratory tract diseases” in Korats (23%), “tumors” in Turkish vans (13%), and “parasites and protozoans” in Cornish Rexes and non-pedigree cats (12 and 36%, respectively). In addition, there was a relatively high prevalence of cardiac issues in Sphynxes (8%), urinary system disease in Abyssinian–Ocicat–Somali (21%), and musculoskeletal condition in Korats (16%), as well as a low prevalence of female genital diseases in non-pedigree cats (5%) compared with all the pedigree breeds (19%) (Figures 4 and 5).

#### Prevalence of Diseases

**Figure 6:**
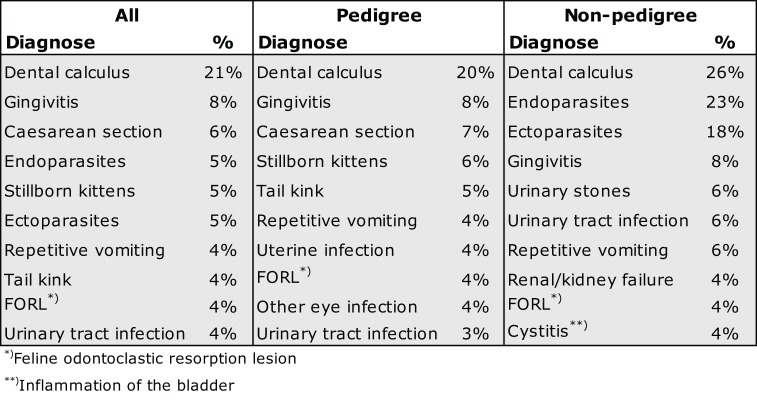
**The 10 most prevalent diseases among all cats, pedigree cats, and non-pedigree cats**.

Dental calculus (21%) was the most prevalent disease among all cats and breeds, except in the age group of <1 year and in the breed group of Cymric–Manxes and the breed of Korats (Figure 6 and Data Sheet S3 in Supplementary Material). The prevalence between breeds varied from 12% in Devon Rexes to 28% in the breed group of Siamese–Balinese–Oriental–Seychellois. Common diseases for all cats and in the majority of the breeds, such as gingivitis (all cats 8%), repetitive vomiting (4%), tail kink (4%), feline odontoclastic resorption lesion (FORL) (4%), and urinary tract infection (4%), were also included. Among pedigree female cats, cesarean section (7% female) and stillborn kittens (6% female) belonged to the most prevalent issues throughout the breeds, whereas among the non-pedigree cats, the prevalence of genital disorders was comparably low.

##### Age

Examples of increasing prevalence from the youngest to the oldest age group were found in dental calculus (<1 year: 0%; ≥11 years: 43%), gingivitis (<1 year: 0%; ≥11 years: 13%), FORL (<1 year: 0%; ≥11 years: 11%), renal/kidney failure (<1 year: 0%; ≥11 years: 16%), urinary tract infection (<1 year: 1%; ≥11 years: 9%), urinary stones (<1 year: 0%; ≥11 years: 7%), and mammary tumor (<1 year: 0%; ≥11 years: 6%). Also, the same tendency was distinct in asthma (<1 year: 1%; ≥11 years: 6%). In all these diseases, the difference between the youngest and the oldest age groups was significant (chi square *p* < 0.0001).

##### Alive and Dead

In most diseases, the prevalence of deceased cats was higher than in alive cats, with the most prominent differences in FIP (alive 0% and dead 11%, chi square *p* < 0.0001) and renal/kidney failure (alive 1% and dead 14%, chi square *p* < 0.0001). Tumors were also overrepresented among deceased cats, particularly in the groups of other (unidentified) tumors (alive 0% and dead 9%, chi square *p* < 0.0001) and the mammary tumor (alive 0% and dead 7%, chi square *p* < 0.0001). Some tumors were represented among deceased cats only, such as the digestive tract tumor (dead 4% and alive *n* = 0) and liver tumor (dead 1% and alive *n* = 0).

As noted above, higher prevalence of FIP was found in deceased than alive cats, but it was also overrepresented in non-neutered than neutered cats (3 vs. 1%, respectively), whereas in most of the other diseases, the order of prevalence between the alive and deceased cats as well as the neutered and the non-neutered cats was reversed. FIP was also the most prevalent disease among cats aged <1 year (5%).

##### Disease Categories

When separate disease categories were considered, the most prevalent diseases were moderate seborrhea (3%) in “dermatological/glandular diseases,” dental calculus and gingivitis (21 and 8%, respectively) in “dental and oral diseases,” renal failure and urinary tract infection (3 and 4%, respectively) in “diseases of the urinary system,” tail kink (4%) in “diseases of the musculoskeletal system,” repetitive vomiting (4%) in “diseases of the digestive tract,” asthma and respiratory infection (3% each) in “diseases of the respiratory tract,” cesarean section and stillborn kittens (6 and 5%, respectively) in the “female genital diseases,” undescended testis (2%) in the “male genital diseases,” and food allergy (3%) in the “endocrine and metabolic diseases.”

##### Individual Breeds

The most prevalent disorder among all breeds, except Korats and the breed group of Cymric–Manxes, was dental calculus (prevalence 21% for all cats). In turn, among Korats, the most prevalent disease was asthma (19%), and in breed group of Cymric–Manxes, variations in the length of the tail (entirely tailless 34%, stumpy 21%). Among the exceptionally high prevalence compared with other breeds, to mention some, were gingivitis in the breed group of Siamese–Balinese–Oriental–Seychellois (18%), sequester of cornea (5%) in the breed group of Persian–Exotic, renal failure (8%) in Korats, protozoans (11%) and HCM (6%) in Sphynxes, stillborn kittens (female 16%) in the breed group Burmese–Burmilla–Singapura, cesarean section (female 15%) in Birmans, different tumors in Turkish vans, and endo- and ectoparasites (23 and 18%, respectively) in non-pedigree cats.

##### Phylogenetic Conditions

**Figure 7:**
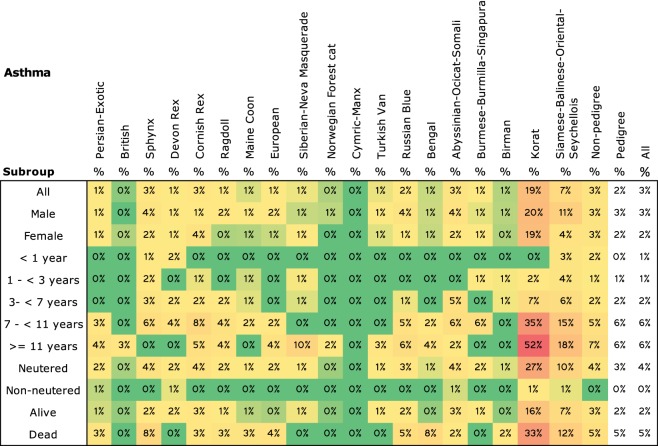
**Prevalence of asthma in all breeds**. Prevalence are colored with gradient filling from the lowest value to the highest (low = green, high = red) to point out the differences between the breeds. Phylogenetic grouping has been used in ordering the breeds.

Distinct phylogenetic connections of the breeds concerning association to the same disease were detected in the anal sac problem (Birman, Korat) (Data Sheet S4 in Supplementary Material), strabismus (Birman, Siamese–Balinese–Oriental–Seychellois), cesarean section (Birman, Burmese–Burmilla–Singapura, Siamese–Balinese–Oriental–Seychellois), and asthma (Korat, Siamese–Balinese–Oriental–Seychellois) (Figure 7). The prevalence of the diseases were higher in these breeds compared with other breeds (non-pedigree cats excluded). Also, the logistic regression analysis verified these breeds associated with the diseases.

### Breed-Specific Diseases

Altogether, 78 diseases were considered tentatively breed-specific, according to disease prevalence and the number of diseased cats (Data Sheets S4 and S5 in Supplementary Material). Fisher’s exact tests’ *p-*values < 0.05 (Data Sheet S6 in Supplementary Material) indicated that in all 78 diseases, there were cat breeds, which were overrepresented compared with other pedigree cats, and in most of the diseases, also compared with non-pedigree cats.

Reflux nephropathy (RN) was proven to be associated with Ragdolls in grounds of Fisher’s exact test *p* value < 0.001, and the fact that Ragdolls were the only breed which had been reported to have had the disease (Data Sheet S4G in Supplementary Material). Of the remaining 77 diseases, altogether 57 diseases were verified to be breed-specific by logistic regression analysis with variables “Age,” “Sex,” and “Alive/dead” included (Data Sheet S7A in Supplementary Material). Also, the variable “Neutered/non-neutered” was considered in the model, but omitted because of a modifying effect with the variable “Age,” and because the exact timing of the neutering with respect to the occurrence of the diseases was not known. Genetic mutation had been already revealed in 6 of the 57 diseases (Data Sheet S7B in Supplementary Material), 32 diseases were suggested as breed-specific conditions (Data Sheet S7C in Supplementary Material), 18 diseases were newly identified as likely breed-specific conditions (Data Sheet S7D in Supplementary Material), and for 21 diseases, breed specificity could not be verified, even though some of them had been proposed to be breed-specific in previous studies (Data Sheet S7E in Supplementary Material).

The diseases with known mutations included polycystic kidney disease (PKD), progressive retinal atrophy (PRA), HCM, and three types of malformations of tail (Table S7B in Supplementary Material). In all these diseases, the high values of MOR and evidence ratio indicated a distinct association of the breed to the disease. For example, in PKD, both the MOR (13.8 when non-pedigree cat excluded) and the evidence ratio (>10^3^) were very high. From the diseases with known mutations, we found the following disease–breed associations in our study: Persian to PKD, Siamese–Balinese–Oriental–Seychellois and Abyssinian–Ocicat–Somal to PRA, Sphynx and British to HCM, and Cymric–Manx to malformations of the tail. The breeds associated with these diseases in our study were the same as in which the gene mutation was found in all other diseases except in HCM.

**Figure 8:**
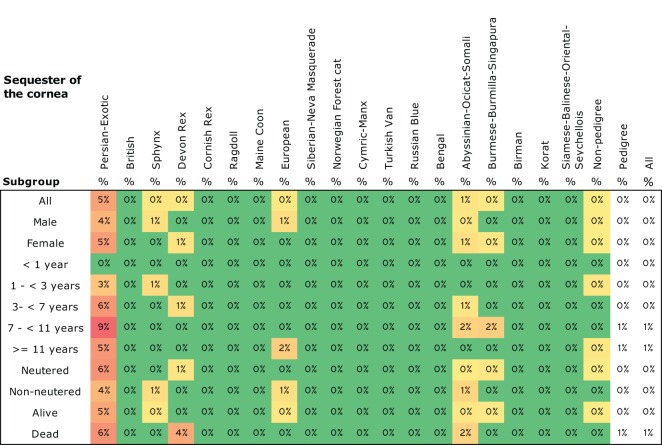
**Prevalence of sequester of the cornea in all breeds**. Prevalence are colored with gradient filling from the lowest value to the highest (low = green, high = red) to point out the differences between the breeds. Phylogenetic grouping has been used in ordering the breeds.

In 18 of the 32 diseases with previously suggested breed-specific conditions, at least one of the breeds found prone in our study was also proven prone in previous studies, but in 14 diseases, breeds in our study and in previous studies differed. The strongest breed-specific associations, according to the MOR and evidence ratios, were found in entropion, sequester of the cornea (Figure 8), hip dysplasia, feline eosinophilic granuloma complex, strabismus (alignment problem of eyes), fungal skin disease, asthma, and malocclusion (teeth alignment problem). All these diseases had MOR over 2.5 and the evidence ratio of over 10^3^. Despite the low value of six of the evidence ratios, restrictive cardiomyopathy (RCM) was considered breed-specific on the grounds of high values of MOR (5.9 non-pedigree cats excluded and 3.9 non-pedigree cats included) and prevalence (Data Sheet S4H in Supplementary Material), which inevitably associated RCM to Cornish Rexes. The low value of the evidence ratio was due to only few cases (*n* = 9) of the disease altogether.

Of the diseases, we found breed-specific for the first time in this study, the best evidence was found for hooked sternum, oligodontia, periodontitis, traumas to the skin, and repetitive constipation with MOR > 2 and the evidence ratio of over 10^3^. Also twisted legs, feline herpesvirus infection (FHV), and ovarian cysts (female) were considered strongly breed-specific, according to high values of MOR (5.4, 3.6, and 2.5, respectively, when non-pedigree cats excluded) and the prevalence, which associated twisted legs to the breed group of Abyssinian–Ocicat–Somali, FHV to Sphynxes and Cornish Rexes, and the breed group of Persian–Exotic to ovarian cysts (Data Sheets S4J,T,N in Supplementary Material, respectively).

We found, altogether, 20 diseases without evidence for breed specificity due to the low values of evidence ratios, indicating that the variable “Breed” did not bring any additional information into the logistic regression models. According to MOR values, some heterogeneity between breeds was observed, but the variation was not large enough to conclude breed specificity.

The OR of breeds proven overrepresented in our study and the references to the previous studies are summarized in Data Sheet S7 in Supplementary Material. Models 1–3 are presented without interactions included, as the interactions did not have a prominent effect on the critical values of our study (MOR, evidence ratio, OR of the breeds) in any of the diseases. Omitting the interactions was also found reasonable because the interaction should have been added in all three models, even in those in which it was not significant, to keep the comparability in counting the evidence ratio (17). Further checking, concerning breeds containing several cats that died before January 1st, 2005 or in which the responses were associated (>15%) with the one breeder, revealed that in fungal skin diseases, most of the cats in the breed group of Cymric–Manx were both associated with the same breeder and had died before January 1st, 2005. No other notable effects caused by cats that died before January 1st, 2005 were found, but other diseases in which one breeder had had an effect to the disease prevalence were detected (see Data Sheet S7A in Supplementary Material).

### Behavioral Abnormalities

We found considerable variation in the behavioral traits between the breeds (Data Sheet S4F in Supplementary Material). For example, 10% of British, Ragdolls, and non-pedigree cats were reported with low activity (not at all or little), whereas the corresponding percentage among all pedigree cats was 5%, and in Bengals – 1%. The British were also reported to have less contact (9% not at all or a little) with people than all cats on average (4%). Turkish vans and Bengals, in turn, were more aggressive toward other cats (15 and 9% aggressive often or very often, respectively) than cats on average (5%). Turkish vans were also reported to have more aggressiveness toward unfamiliar people (6% aggressive often or very often) than cats in all the other breeds (<3% in each). Turkish vans, Russian blues, Bengals, Siamese–Balinese–Oriental–Seychellois, and non-pedigree cats indicated high proportions in sensitivity toward new things (>3% sensitive very often in each breed) as well as toward new people (>4% sensitive very often in each breed). In all pedigree cats, the corresponding proportion was 2% in both traits. Licking was common in many breeds with the highest proportions in non-pedigree cats and in the breed group of Burmese–Burmilla–Singapura (often or very often 8% in both).

In the logistic regression models, breed was an essential variable in the models in terms of evidence ratio (>10^3^) (Data Sheet S7F in Supplementary Material) in all other behavioral traits, except in “aggressiveness toward family” and “contact with people.” In “contact with people” the value of evidence ratio (60) indicated a still relatively important role for breed in the model, but in “aggressiveness toward family,” a lower value of AIC in the model without the breed indicated that the model without a variable breed was better than with the breed included. The highest MOR was in “aggressiveness toward strangers” with the Turkish van as being the most aggressive breed.

## Discussion

The implementation of genetic research requires knowledge of breed-specific health issues to decide the best strategies for patient recruitment. Our feline health survey in a population of over 8000 cats in Finland provides a comprehensive overview of the health profiles and not only forms a strong foundation for genetic research but also gives useful information to breeders, cat fanciers, and veterinarians. This study covered 31 breeds and a large non-pedigree cat population as an appropriate reference group to pedigree cats. The living conditions of non-pedigree cats, such as environment, outdoor activities, and treatment, closely resembled those of pedigree cats (data not shown). In addition, the high percentage of non-neutered cats suggests that the owners are equally conscious as the owners of pedigree cats. The study explored the prevalence in altogether 227 separate diseases identifying the common health issues in cats and 57 breed-specific conditions as well, that can be targeted for genetic research.

### Disease Prevalence and Common Diseases

The most common disease categories and individual diseases found in our study have also been described in earlier studies. Dental diseases were the most prevalent category in the UK study (20), and dental calculus and gingivitis the most prevalent diseases in a US study (21). Dermatological diseases were among the most prevalent disease categories in UK, Japanese, and Swedish studies (20, 22, 23). Furthermore, diseases of the urinary system and digestive tract (22, 23) and ophthalmological diseases (22) are common categories.

The list of common individual diseases include dental calculus and gingivitis, parasites (21), vomiting (20, 21, 23), urinary tract infection (20), kidney disorders (20, 21), urinary stones (23), diarrhea (20, 21), and cystitis (21, 23). Although many of these were found in our study, there are also differences that can be explained by the study design (population vs. veterinary data) and size, geographical region, disease definitions, and data source. For example, the data in the US (*n* = 14 270) (21) and UK (*n* = 3584) (20) studies were based on veterinary clinical data, while the data from Sweden (*n* = 301 485) (23) and Japan (*n* = 49 450) (22) comes from an insurance database; ours is population data.

Higher morbidity of male cats to common diseases (in early age) has been found in Sweden (23). The higher morbidity of male cats was rationalized by anatomical (urinary system) and behavioral (causing traumas) differences between male and female cats. The prevalence in the category of “diseases of the urinary system” was also higher for male cats in our study. Traumas were not treated like a disease category in our study, as in Swedish study, but male cats had significantly more traumas to the skin (chi square *p* = 0.0066) than female cats. However, omitting the diagnoses of urinary tract diseases and skin traumas, we found a higher morbidity rate in males. This is due to the fact that male cats were also overrepresented in a majority of the other disease categories. In the Japanese and Swedish studies (22, 23), domestic and cross-bred cats had been stated to be more prone to accidents, and the assumed cause was different access to outdoors. In our study, the most overrepresented breed in skin traumas was non-pedigree cats, which roamed outside more than pedigree cats (data not shown), but also, breed groups of Siamese–Balinese–Oriental–Seychellois and Abyssinian–Ocicat–Somali and Norwegian Forest cats were overrepresented, as well. The different outdoor activities did not explain their higher prevalence of traumas. The more frequent outside roaming was, instead, the most probable cause of higher prevalence of parasites among non-pedigree cats compared to pedigree cats.

Our results suggest that non-pedigree cats have more diseases than pedigree cats with a higher percentage of non-pedigree cats having diseases at least in one of the disease categories (chi square *p* < 0.0001). However, when only veterinary diagnoses were considered, and the disease category of “Parasites” was omitted, the difference was not significant anymore (*p* = 0.0745), even though non-pedigreed cats were still overrepresented compared with pedigree cats. A potential explanation could relate to the differences in the selection of the reported cats between the owners of pedigree and non-pedigree cats (see limitation below). UK and Japan studies also found equal prevalence of disease in pedigree and non-pedigree populations (20, 22).

In many diseases and disease categories, disease prevalence increased with age up to ≥11 years. The increase was especially strong in disease categories of “dental and oral diseases,” “diseases of the urinary system,” and “tumors.” A similar increase in these same disease categories was reported previously in Japan and Sweden (22, 23), although our study found more age-related disease categories. This is because we asked to report the health history to cover the earlier stages of life and not only at the time of reporting. For the same reason, the peak in the youngest age group, which was found in many disease categories in the Japan and Sweden studies (22, 23), remained uncovered in our study. For example, we found that the prevalence of the disease category of “parasites and protozoans” increased cumulatively throughout the age groups, due to inclusion of cured infestations from the earlier years of age (as confirmed from the additional information of the questionnaire), whereas in Japan and Sweden (22, 23), the prevalence of the “parasites and protozoans” category was high only in the first age group.

The disease prevalence in most of the disease categories and diseases were higher in deceased than living cats, and neutered than non-neutered cats. An interesting exception was the prevalence of feline infectious peritonitis (FIP), which was higher among living and non-neutered cats. FIP was also the most prevalent disease in age group of <1 year. The reason why FIP was prevalent in young and deceased cats results from the clinical picture of FIP as an infectious disease of young cats with high mortality (24). The high prevalence among non-neutered cats was due to the age distribution of non-neutered cats with 73% of them being aged <3 years.

Phylogenetic association to a disease, which was detected in the anal sac problem, strabismus, HCM, cesarean section, and asthma, may imply inheritance of the genetic predisposition *via* a common ancestor of the phylogenetic group of cats. Previous studies suggest the same to strabismus (25) and cesarean section (26). In both diseases, the phylogenetically associated breeds in our study, i.e., Birman and Siamese–Balinese–Oriental–Seychellois group, were also found to be associated in the previous studies. Instead, the anal sac problem lacks associations to any breed. Only Anal sac gland carcinoma (ASGC) was found associated with the Siamese breed (27), whereas the breeds found associated with the anal sac problems in our study were Birman and Korat. However, Siamese, Birman, and Korat breeds belong to the same eastern-derived population (14), which might indicate an inherited common predisposition to problems in anal sacs. Asthma was found phylogenetically associated with Korats and the Siamese–Balinese–Oriental–Seychellois group in our study. It has been associated with the Siamese breed in many previous studies also (28–30). No study was found, in which asthma would have been connected to Korats, but association was found to Birmans (31). A common predisposition to asthma by the breeds belonging to the same eastern-derived population could be considered.

### Breed-Specific Disorders

One of our key objectives was to identify potential breed-specific diseases for genetic studies. Breed specificity in diseases often implies heredity (3), and, thus, breed-specific diseases are appropriate candidates to be considered taking into genetic research. We found 58 such diseases, including RN, sequester of cornea, entropion, feline eosinophilic granuloma complex, periodontitis, oligodontia, urinary tract infection, HCM (Sphynxes and British), RCM, hip dysplasia, hooked sternum, asthma, and stillborn kittens. HCM mutation has been found in Ragdolls and Maine Coons (9, 10), but the Sphynxes and British’, which were found overrepresented in our study, have also been associated with HCM in previous studies (32–34). RN was found only in Ragdolls. Of the other diseases mentioned, at least one of the breeds found overrepresented in our study had also been found overrepresented in previous studies, including sequester of cornea (35, 36), entropion (37, 38), asthma (28–30), strabismus (39, 40), and hip dysplasia (41). RCM had been found overrepresented in non-pedigree shorthairs. As potential new breed-specific conditions, we found feline eosinophilic granuloma complex, periodontitis, oligodontia, urinary tract infection, hooked sternum, and stillborn kittens.

However, further studies concerning diseases suggested to be breed-specific in our study must be conducted before proceeding in genetic research. It is essential to also map other contributing factors concerning lifestyle of the cat, such as environment, diet, and outdoor activities, as well as the differences between the breeders and possible cross effects between different diseases. For example, entropion and herpes virus in the eye have been proposed as contributing factors in sequester of cornea (42). Also, our data confirmed the association between sequester and both of these diseases (Fishers’ exact test *p* < 0.001 for both when cats aged <1 year excluded), but the major part of the sequester cases did not have either of these associations, also allowing other possible triggers for sequester as well as for entropion. Also, nystagmus and malocclusion, which were found to be breed-specific in our study, have been proposed to be symptoms of other diseases or physical features rather than independent disorders (35, 38, 43). Ongoing studies will investigate the possible associations of above-mentioned factors to common and breed-specific disease predisposition.

In diseases in which no breed specificity was detected in our study, digestive tract tumor (44), birthing complications (26, 45), FIP (46–48), and pyometra (49) were found to be breed-specific in previous studies. The reason for this was the low variation of prevalence between the breeds, which might be due to underreporting in certain breeds, diagnostic problems, or different environmental factors compared with the other studies.

### Behavioral Variation

In the preliminary analysis of behavioral traits, most divergent behavior was found in British, Ragdolls, Turkish Vans, Korats, and Bengals. Both British and Ragdolls (along with non-pedigree cats) were the most inactive breeds, and, in addition, British had less contact with people than cats on average (Data Sheets S4 and S7 in Supplementary Material). Ragdolls have been described as less active earlier (50). Turkish Vans were distinctively the most aggressive breed toward strangers and other cats. Korats also showed aggression toward family members and unfamiliar people, and Bengals – toward other cats. This finding in Bengals has been observed before (50). We also found variation in sensitivity and licking in few breeds. Overall, these results encourage a more comprehensive future behavioral study in cats to establish proper genetic study cohorts and to identify environmental contributors.

### Limitations

Health surveys targeted to pet breeders and owners have limitations. One of the key concerns is related to the responder distribution in the population since the cat owners were reached by information channels which might have favored breeders and other cat fanciers over owners who own cats as pets but are not really cat fanciers. This may have resulted in non-response error by some cat owners not participating in the study and in measurement error by some responders giving inaccurate or incomplete information (51). Suspicions like these have been also expressed in meetings with the cat breeders and owners during the study to encourage reports from both healthy and affected cats. Inaccurate and incomplete information may have been caused by misunderstandings or missing some diagnoses leading to under- or overestimates of prevalence, especially if the owner had made the diagnosis. Therefore, we have reported prevalence data in various diagnostic groups to allow comparisons.

The third limitation may relate to the scarcity of Finnish breeders or the large proportion of breeders not participating in some breeds, which may have resulted in a concentration of responses only from a few breeders. This might have led to bias, especially in infectious diseases, making a local epidemic seem like a high prevalence in the whole breed. These kinds of effects were sought during the study, and if signs of such concentration were detected, they were marked in the Table S7 in Supplementary Material. It is important to also keep in mind that Finnish cat breeds may differ genetically from breeds in other countries because of differences in breeding practices, which can create distinct lines within a single breed (4). This may have caused genetic concentration or dilatation in Finnish breeds, yielding deviant prevalence.

Also, the design of our study, with disease occurrences including earlier states of life in addition to the time of submitting the questionnaire, gives ground to presume prevalence in our study to be higher than in prevalence studies in which only occurrences of a certain point of time are accepted. In addition, it is appropriate to point out that logistic regression yields overestimates of OR when the prevalence of the disease is greater than 5% (15). However, in our study, logistic regression was targeted to designate the effect of the whole variable “Breed” by means of MOR, and the yielded OT in case of prevalence >5% in Model 3 may be considered suggestive of the approximate magnitude of the effects of separate breeds, not the exact value of odds ratio.

We also recognize that “food allergies” was suboptimally placed in the category of “endocrine and metabolic diseases” instead of the categories of digestive tract diseases. This may have caused overestimates in the prevalence of the category of endocrine and metabolic diseases. However, it is more important to pay attention to the prevalence of specific conditions rather than broader categories.

Despite all suggested reasons causing possible bias, prevalence in our study match well with the results stated in earlier studies and are underestimates rather than overestimates in case of disparity giving conservative inferences (20, 21). It is more likely that some breed-specific diseases have been missed rather than breed specificity has been suspected in vain.

## Conclusion

This unique study gives valuable prevalence information of 227 feline diseases in Finnish cats and identifies nearly 60 breed-specific disorders that can be targeted for genetic characterization. Compared to previous studies, we provide the broadest countrywide health survey available so far with designed breed-specific sample size requirements. The study established a health survey platform and database, which has value not only for genetic research programs but also provides for veterinary medicine and breeding programs.

## Author Contributions

HL conceived and designed the study with KV and A-MV. KV designed and performed statistical analyses with the help from A-MV. HL, KV, TJ, A-MV, KT, and JT contributed to the development of the questionnaire and material collection. KV and HL drafted the manuscript with the help from others.

## Conflict of Interest Statement

The authors declare that the research was conducted in the absence of any commercial or financial relationships that could be construed as a potential conflict of interest.

## Footnotes

^1^http://www.cfa.org/

^2^http://www.tica.org/

^3^http://www.kissaliitto.fi/

^4^www.kissangeenit.fi
