# Supplementary material for: Health and Behavioral Survey of over 8000 Finnish Cats
Source: Front Vet Sci. 2016 Aug 29;3:70. doi: 10.3389/fvets.2016.00070 (PMC5002895; doi:10.3389/fvets.2016.00070)
Supplement: Supplementary file 1 [file Presentation_1.PDF]

Feline Health Questionnaire

This study is carried out by the University of Helsinki's Canine and Feline Genetic Research group. The purpose of this study is to map the incidence of illnesses experienced by cats residing in Finland. Through this questionnaire, we want to research permanant illnesses as well as temporary illnesses also from which the cat has regained health. **It is very important for owners to fill out questionnaires for healthy cats as well!** This way we can have a realistic picture of the health situation of the entire cat breed. It is also important to receive results for already deceased cats, so that we can understand the most common causes of death.

This health questionnaire benefits every cat owner and breeder, as the knowledge and understanding of common feline illnesses may give us the opportunity to prevent and address the problem. If we want to breed healthy and thriving cats, it is essential to understand the current healthy situation of each cat breed and cats as an entire species. The results from the analysis of this questionnaire will help in the research of genetic illnesses and characteristics. If we receive many answers, based on the this material we can perform new research on certain illnesses. Another goal behind this health questionnaire is to create a health database for each breed, where the owner can update their own cat (s) information by themself. In this way the owner would also be able to acquire the most current information for breeding assistance.

Individual owners or cats will not be identified in any publicated research. The credibility of the research and mapping of feline illness depends on the number of thoroughly filled out questionnaires total. Every completed questionnaire is valuable regardless of the cat's current health status!

If you have several cats, please fill out a separate questionnaire for each one. Save the completed questionnaire and open a new one for the next cat.

Before beginning to complete the questionnaire, we recommend you take out your cat's registration papers and health information beforehand!

ATTENTION! Results will be handled with full **confidentiality.** The information regarding each cat and owner will be saved to a classified database to which only members of the research group have access. These include student Tara Joensuu, statistician Katariina Vapalahti, professor Hannes Lohi ja docent Anna-Maija Virtala.

Any questions regarding the questionnaire or updates or corrections to a previously filled out questionnaire can be addressed to lgl-kyselyt@helsinki.fi. Thank you very much for your interest in our research.

Mandatory questions are marked with an asterisk (*).

1. Cat Information

1. Breed of Cat

Other breed:

2. Cat's registered name ?

3. Cat's nickname

Registration number is required! If your cat does not have a registration number, please write ¨home cat¨ or ¨I don't have access¨.

4. Registration number

5. EMS-code

6. Birthday (day.month.year example 03.02.2005)

7. Date of death if applicable (day.month.year)

8. Cause of death?

	Yes	No	I don't know
**9. Have you given a blood sample from your cat to professor Hannes Lohi's cat DNA-bank?**			

	Male	Female
**10. Gender**		

	Yes	No	Where?
**11. Has your cat been born outside of Finland?**			

	Yes	No	If so, when and for how long?
**12. Has your cat been surgically castrated/sterilized?**			
**13. Has your cat had a hormone implant?**			
**14. Has your cat used birth control pills?**			

	A	B	AB	I don't know.
**15. Blood group**				

16. Cat's weight

17. Estimate your cat's weight

	Very underweight	A little underweight	Ideal weight	A little overweight	Very overweight
**My cat is**					

	I don't know	Under 8 weeks old	8-9 weeks old	10-11 weeks old	12-13 weeks old	14-15 weeks old	16 weeks- 1 year old	Adult	Never separated from the mother
**18. At what age has your cat been weaned from his/her mother? (the age of physical separation)**	○	○	○	○	○	○	○	○	○

	From birth	Under 9 weeks old	10-11 weeks old	12-13 weeks old	14-15 weeks old	16 weeks- 1 year old	As an adult	I don't know
**19. At what age has the cat come to you?**	○	○	○	○	○	○	○	○

20. Father's official name

Father's registration number

21. Mother's official name

Mother's registration number

2. Owner Information

1. Owner's name
2. Email
3. Address
4. Region
5. Phone number

We will contact you only in case of questions regarding the questionnaire.

	Male	Female
**6. Owner's gender**	○	○

	Under 18	18-25	26-35	36-45	46-55	56-65	66-75	Over 75 years
**7. Owner's age**	○	○	○	○	○	○	○	○

8. Are you this cat's breed's registered breeder?

Yes	No	If you answered ¨Yes¨, how many litters have you raised?
○	○	

3. Cat's Descendants

	Yes	No	I don't know
**1. In the future, will you use this cat for breeding?**	○	○	○

	Yes	No	I don't know
**2. Does this cat have descendants?**	○	○	○

3. How many litters has your cat had in total?
4. How many kittens has your cat had in total that have survived at least 2 months?
5. In total, how many kittens have been stillborn?
6. How many kittens died prior to weaning (however not stillborn)?

How/why have the kitten(s) died?

4. Environment and Diet

	Indoors	Outdoors	Mainly indoors, but can go outside	Mainly outdoors, but can come inside
**1. Where does the cat live?**	○	○	○	○

	Outside the entire time	Every day	3-5 times per week	1-2 times per week	1-2 times per month	A few times per year	Never
**2. How often does your cat go outdoors?**	○	○	○	○	○	○	○

3. If your cat is only indoors (never goes outdoors), estimate the size of the the living quarters
4. How does your cat typically go outdoors?

☐ In a confined area/garden or balcony

☐ On a leash

☐ Free to roam

☐ None of the above (answer in the text box below!)

Tell us more:

5. IF your cat goes outside, does he/she

	Yes	No	What and how often?
**bring prey inside the house (mice, voles, birds etc)**	○	○	
**bring prey into the yard?**	○	○	
**eat the prey?**	○	○	
**give you the prey as a ¨gift¨?**	○	○	

6. Does your cat live with other animals? If yes, which species?

☐ Cat(s)

☐ Dog(s)

☐ Pet bird(s)

☐ Rodents (rabbits, guinea pigs, hamsters)

☐ Fish

☐ Domestic animal (sheep, cows, chicken etc)

☐ Other species

How many other animals live in the same household? Number : [ ]

	1 adult	2 adults	3 adults	4 adults or more
**7. How many adults live in your household?**	○	○	○	○

	0 children	1-2 children	3-4 children	5-6 children	7 children or more
**8. How many children live in your household?**	○	○	○	○	○

9. What does your cat mainly eat?

☐ Industrial dry/wet food (bought from the grocery store)

☐ Industrial dry/wet food (bought from the pet store)

☐ Raw fish or meat stored in the freezer

☐ Raw fish or meat not stored in the freezer

☐ Cooked meat/fish

☐ Home food made specifically for the cat

☐ Home food: human food leftovers

Other food, what? [ ]

	Yes	No
**10. Is the food accessible to the cat the entire day?**	○	○

	Yes	No	What and how often?
**11. Does your cat receive continuously or regularly nutritional supplements (for example vitamins, oil)?**	○	○	[ ]

	Yes	No
**12. Does your cat have a scratching post (or own designated place where he/she can sharpen claws)?**	○	○
**13. Does your cat have the possibility to sleep up high?**	○	○

5. Personality and Behavior

1. Personality

	Not at all	A little	Quite a bit	Often	Very often	More information
**Activity level**	○	○	○	○	○	
**Tries to take contact with people**	○	○	○	○	○	
**Aggressiveness towards strangers (people)**	○	○	○	○	○	
**Aggressiveness towards own family members**	○	○	○	○	○	
**Aggressiveness towards other cats**	○	○	○	○	○	
**Sensitivity/shyness towards new situations**	○	○	○	○	○	
**Sensitivity/shyness towards strangers (people)**	○	○	○	○	○	
**Licking/biting/sucking self**	○	○	○	○	○	

	Yes	No	If so, please describe
**2. Is there loss of fur due to licking?**	○	○	
**3. Separation anxiety behaviour?**	○	○	
**4. Overly active behaviour?**	○	○	

5. How often does your cat knead?

6. Does your cat show any of the following behaviour? How often?

	Never	1-3 times in their life	1-12 time per year	1-4 times per month	1-3 times per week	Every day	Many times per day	Most of the time per day
**Biting/sucking of cloth or plastic**	○	○	○	○	○	○	○	○
**Spraying indoors**	○	○	○	○	○	○	○	○
**Bowel movements outside of the catbox**	○	○	○	○	○	○	○	○
**Aggression towards self**	○	○	○	○	○	○	○	○
**Continuous meowing/other sound**	○	○	○	○	○	○	○	○
**Hyperesthesia Syndrome (cat behaves as though skin is ticklish, sore, mite bite its own tail)**	○	○	○	○	○	○	○	○

**Tail chasing or spinning**	◯	◯	◯	◯	◯	◯	◯	◯
**Unexplainable frightened behaviour**	◯	◯	◯	◯	◯	◯	◯	◯
**Cat seems to lose connection with the environment/loses touch with reality**	◯	◯	◯	◯	◯	◯	◯	◯

	Yes, diagnosed by the vet	Yes, self-diagnosis	No	I don't know.
**7. Does your cat have a behavioural problem or other tendency?**	◯	◯	◯	◯

Tell us more about the behavioral problem (when it began, in what situations it appears etc), or describe if your cat has another odd habit

6. Gene tests and Ultrasounds

1. If your cat has undergone genetic screening, what were the results?

	Healthy	Carrier (one copy)	Sick (double copy)	I don't know/Hasn't been tested
**PKD-gene test**	◯	◯	◯	◯
**GM1**	◯	◯	◯	◯
**GM2**	◯	◯	◯	◯
**rdAc-PRA**	◯	◯	◯	◯
**PK**	◯	◯	◯	◯
**SMA**	◯	◯	◯	◯
**GSD IV (glycogen storage disease type IV)**	◯	◯	◯	◯
**HCM Ragdoll (hypertrophic cardiomyopathy)**	◯	◯	◯	◯
**HCM Maine Coon (hypertrophic cardiomyopathy)**	◯	◯	◯	◯
**Hypokalemia**	◯	◯	◯	◯

Other genetic test and its result:

Answer these questions IF your cat has had an ultrasound of the heart, kidney or other organ.

	Healthy	Borderline case	Sick	I don't know/Hasn't been tested	At what age?
**2. What were the latest ultrasound results for HCM?**	○	○	○	○	
**3. What were the latest heart ultrasound results for DCM?**	○	○	○	○	
**4. What were the latest heart ultrasound results for RCM?**	○	○	○	○	
**5. What were the latest kidney ultrasound results for PKD?**	○	○	○	○	
**6. What were the latest kidney ultrasound results for refluxive nephropathia?**	○	○	○	○	

	Yes	No	I don't know	If you answered "Yes", why and when?
**8. Has your cat undergone structural radiological investigations?**	○	○	○	

	Yes	No	I don't know	If you answered ¨yes¨, at what age?
**8. Has your cat had an ultrasound for another reason (other than pregnancy)?**	○	○	○	

7. Congenital developmental disorders

	Yes, diagnosed by the vet	Yes, self-diagnosis	No	I don't know.
**Has your cat been diagnosed with a congenital birth defect?**	○	○	○	○

Please indicate the illness/problem below:

- ☐ Congenital hypotrichosis
- ☐ Congenital small eyes (microphtalmia)
- ☐ Congenital heart disease (VSD etc)
- ☐ Cleft palate
- ☐ Portosystemic shunt
- ☐ Spina bifida
- ☐ Omphalocele (BE: Omphalocoele)
- ☐ Developmental or growth problem of the kidneys
- ☐ Other illness/problem (Attention! Please specify below in the textbox)

Please provide more information regarding the illness/problem (when it was diagnosed, medication, age of onset, symptoms etc)

8. Dermatological/glandular diseases

	Yes, diagnosed by the vet	Yes, self-diagnosis	No	I don't know.
**Has your cat been diagnosed with a skin-related problem or illness?**				

Please indicate the illness/problem(s) below:

- Trauma to the skin (burn, bite, frostbite etc.)
- Skin allergy
- Skin infection/inflammation
- Abnormal skin pigmentation/inflammation (lentigo, vitiligo etc.) (ATTENTION! Clarify in the textbox)
- Fungal skin disease (ringworm, yeast infection, sporotrichosis, cryptococcosis)
- Bacterial skin disease
- Seborrea requiring medical care (acne, stud tail etc.)
- Moderate (doesn't require medicine) Seborrea (acne, stud tail, overactive sebaceous glands)
- Pathological loss of fur (alopecia jne.)
- Anal gland problem
- Superficial pyoderma (wound infections, impetigo)
- Feline cutaneous astenia (fragile/loose skin caused by abnormal collagen production)
- Feline Eosinophilic granuloma complex (lesions on thigh, swelling on face etc.)
- Feline ulcerative dermatitis (excessive hair loss and scabbing)
- Chediak-Higashi syndrome
- Claw-related illness/symptom (ATTENTION! Clarify in the textbox)
- Other fur-related illness/symptom (ATTENTION! Clarify in the textbox)
- Other skin-related illness/symptom (ATTENTION! Clarify in the textbox)

Please provide more information regarding the illness/problem (when it was diagnosed, medication, age of onset, symptoms etc)

9. Ocular diseases

Cat's eye colour

	Yellow	Green	Blue	Other colour	If you choose another colour, which colour?
Right eye	○	○	○	○	
Left eye	○	○	○	○	

	Yes, diagnosed by the vet	Yes, self-diagnosis	No	I don't know.
Has your cat been diagnosed with a eye-related problem or illness?	○	○	○	○

Please indicate the illness/problem(s) below:

- ☐ My cat is blind
- ☐ Eye infection caused by Herpesvirus
- ☐ Other eye infection
- ☐ Dermoid cyst
- ☐ Glaucoma (eye pressure disease)
- ☐ Strabismus
- ☐ Sequester of the cornea
- ☐ Illness/problem due to trauma (bite, burn etc.)
- ☐ Other illness/problem (Attention! Please specify below in the text box)

Tear gland & tear duct

- ☐ Cherry eye
- ☐ Blocked or missing lacrimal duct
- ☐ Conjunctival infection
- ☐ Another lacrimal gland/duct illness/problem (ATTENTION! Please clarify in the text box below)

Eyelid-related illnesses and problems

- ☐ Entropion (eyelid abnormally turned inwards)
- ☐ Extra fold in the temporal corner of eyelid
- ☐ Protrusion of the third eyelid
- ☐ Other eyelid-related illness/problem (ATTENTION! Please clarify in the text box below)

Lens-related illness/problem

- ☐ Cataract
- ☐ Lens luxation
- ☐ Other lens-related illness/problem (ATTENTION! Please clarify in the text box below)

Retina-related illness/problem

- ☐ Retinal degeneration

☐ Progressive retinal atrophy (PRA)

☐ Other retinal illness/problem (ATTENTION! Please clarify in the text box below)

Please provide more information regarding the illness/problem (when it was diagnosed, medication, age of onset, symptoms etc)

10. Otic diseases

	Yes, diagnosed by the vet	Yes, self-diagnosis	No	I don't know
**Has your cat been diagnosed with a ear-related problem or illness?**	○	○	○	○

Please indicate the illness/problem(s) below:

☐ My cat is deaf (Attention- Additional questions below!)

☐ Repetitive outer ear infection(otitis externa)

☐ Repetitive inner ear infection (otitis interna)

☐ Ear hematoma (not caused by trauma)

☐ Other illness/problem (ATTENTION! Please clarifyin the text box below)

Please provide more information regarding the illness/problem (when it was diagnosed, medication, age of onset, symptoms etc)

Deaf cat's Baer-test

	Yes	No	I don't know
**2. Has your cat had a Baer-test?**	○	○	○
**3. Is your cat deaf?**	○	○	○
**4. Is your cat white?**	○	○	○

5. IF your cat is deaf

	Both ears	Right ear only	Left ear only	I don't know/Cat isn't deaf
**Is your cat deaf in one ear or both?**	○	○	○	○

11. Dental and oral diseases

	Yes, diagnosed by the vet	Yes, self-diagnosis	No	I don't know
**Has your cat been diagnosed with a mouth or teeth-related problem or illness?**	○	○	○	○

Please indicate the illness/problem(s) below:

- ☐ Malocclusion (teeth alignment problem)
- ☐ Gingivitis (inflammation of gum tissue)
- ☐ Stomatitis (inflammation of mouth and lips)
- ☐ Periodontitis (soft tissue gum infection)
- ☐ Feline odontoclastic resorption lesion (FORL, feline cavity, neck lesion)
- ☐ Dental calculus
- ☐ Tooth fracture
- ☐ Oligodontia
- ☐ Other illness/problem (ATTENTION! Please clarify in the text box below)

Please provide more information regarding the illness/problem (when it was diagnosed, medication, age of onset, symptoms etc)

12. Diseases of the urinary system

	Yes, diagnosed by the vet	Yes, self-diagnosis	No	I don't know
**Has your cat been diagnosed with a kidney or urinary tract-related problem or illness?**	○	○	○	○

Please indicate the illness/problem(s) below:

- ☐ Cystitis (inflammation of the bladder)
- ☐ Idiopathic cystitis
- ☐ Feline urologic syndrome
- ☐ Urinary incontinence (difficult to hold urine)
- ☐ Urinary tract infection
- ☐ Urinary stones in lower urinary tracts
- ☐ Other illness/problem (ATTENTION! Please clarify in the text box below)

Munuaistauti/Nefropatia

- ☐ RN (refluxive nephropathy)
- ☐ Kidney infection (nephritis)
- ☐ Nephrosis (degenerative disease of the renal tubules; hydronephrosis ja amyloid nephrosis)
- ☐ Uremia (and azotemia)
- ☐ Renal/kidney failure
- ☐ PKD (Polycystic Kidney disease)
- ☐ Glomerulonephritis

Kidney stones

Please provide more information regarding the illness/problem (when it was diagnosed, medication, age of onset, symptoms etc)

13. Disorders of the cardiac and circulatory system

	Yes, diagnosed by the vet	Yes, self-diagnosis	No	I don't know
**Has your cat been diagnosed with a heart or circulation-related problem or illness?**				

Please indicate the illness/problem(s) below:

- HCM (hypertrophic cardiomyopathy)
- RCM (restrictive cardiomyopathy)
- DCM (dilated cardiomyopathy)
- SAS (subaortic stenosis)
- High blood pressure (hypertension)
- Blood vessel blockage (thrombosis and thromboembolism)
- Unidentified heart murmur
- Other illness/problem (ATTENTION! Please clarify in the text box below)

Please provide more information regarding the illness/problem (when it was diagnosed, medication, age of onset, symptoms etc)

14. Blood disorders

	Yes, diagnosed by the vet	Yes, self-diagnosis	No	I don't know
**Has your cat been diagnosed with a blood-related problem or illness?**				

Please indicate the illness/problem(s) below:

- Anemia
- Bloody nose (not caused by trauma)
- Acquired circulatory illness/disorder (due to trauma, vaccinnation or poisoning etc)
- FIA (feline infectious anemia)
- Blood disorder related deficiency (Example. Von Willebrand's disease, hemophilia A/B, Factor X/XI/XII, vitamin-K etc.) (ATTENTION! Please clarify in the text box below)

☐ Blood platelet related (thrombocytopenia) (ATTENTION! Please clarify in the text box below)

☐ White blood cell related idiopathic illness/problem (leukopenia, lymphocytosis, eosinophilia etc.) (ATTENTION! Please clarify in the text box below)

☐ Abnormal level of lipids or lipoproteins in blood

☐ Other circulatory system-related illness/problem (ATTENTION! Please clarify in the text box below!)

Please provide more information regarding the illness/problem (when it was diagnosed, medication, age of onset, symptoms etc)

15. Diseases of the musculoskeletal system

	Yes, diagnosed by the vet	Yes, self-diagnosis	No	I don't know
**Has your cat been diagnosed with a skeletal-related problem or illness?**	○	○	○	○

Please indicate the illness/problem(s) below:

☐ Traumatic accident (traffic accident, fall from height, bone fracture etc.)

☐ Osteoarthritis/degenerative joint disease

☐ Hip dysplasia

☐ Bone or cartilage developmental disorder (osteochondrodysplasia, achondroplasia)

☐ Bone growth disorder, dwarfism (achondroplasia)

☐ Umbilical hernia

☐ Diaphragmatic hernia

☐ Other hernia

☐ LIgament damage

☐ SMA (spinal muscular athrophy)

☐ Muscle weakness (Myopathy; Duchenn's & Becker's dystrophy etc)

Malformations (also characteristic to a particular breed)

☐ Tail kink (crooked/bent tail)

☐ Entirely tailless (rumple/dimple rumpy)

☐ Riser/ rumpy riser /stumpy

☐ Stubby (8 cm)

☐ Hooked sternum

☐ Dorsoventral flattening of the sternum (also as kittens)

☐ Hallowed chest (pectus excavatum)

- Oligodactylia
- Toe deformity
- Twisted legs
- Frog-legged (also as a kitten)
- Ringtail

Luxations

- Patella luxation
- Elbow luxation
- Shoulder luxation
- Other joint luxation

Other muscle-/skeletal-related illness/problem

Please provide more information regarding the illness/problem (when it was diagnosed, medication, age of onset, symptoms etc)

16. Diseases of the digestive tract

	Yes, diagnosed by the vet	Yes, self-diagnosis	No	I don't know
**Has your cat been diagnosed with a digestive system-related problem or illness?**				

Please indicate the illness/problem(s) below:

- Repetitive vomiting including hairballs (Please write in the tex box HOW OFTEN)
- Repetitive constipation (clarify in the text box HOW OFTEN)
- Repetitive diarrhea (clarify in the text box HOW OFTEN)
- Enlarged esophagus (Megaesophagus)
- Enlarged colon (Megacolon)
- Exocrine pancreatic insufficiency (EPI)
- Intestinal blockage

☐ Intussusception

☐ Jaundice/icterus

Infections

☐ Esophagitis (inflammation of esophagus)

☐ Pancreatitis (inflammation of pancreas)

☐ Acute inflammatory bowel disease

☐ Chronic inflammatory bowel disease

☐ Gastritis (Gastric Inflammation)

Liver related illnesses/problems

☐ Hepatitis (liver infection, all forms) (ATTENTION! Please clarify in the text box below!)

☐ Cholangiohepatitis (liver & bile duct infection)

☐ Fatty liver, hepatitis lipidosis

☐ Wilson's disease (collection of copper in the liver)

Other illness/problem related to the digestive system:

Please provide more information regarding the illness/problem (when it was diagnosed, medication, age of onset, symptoms etc)

17. Diseases of the respiratory tract

	Yes, diagnosed by the vet	Yes, self-diagnosis	No	I don't know
**Has your cat been diagnosed with a respiration-related problem or illness?**	○	○	○	○

Please indicate the illness/problem(s) below:

☐ Asthma

☐ Dyspnea (shortness of breath)

☐ Chronic cough

☐ Throat infection (Is it a tonsil, larynx or other infection? Please clarify in the text box below)

☐ Nasal mucus membrane infection (rhinitis)

☐ Respiratory infection

☐ Bronchitis (bronchi mucus membrane infection)

☐ Lung infection

☐ Liquid accumulation in the pleural cavity (pleural effusion, hydrothorax)

☐ Chylothorax (lymph fluid accumulation in pleural cavity)

☐ Other respiratory system-related illness/problem (ATTENTION! Please clarify in the text box below.)

Please provide more information regarding the illness/problem (when it was diagnosed, medication, age of onset, symptoms etc)

18. Diseases of the nervous system

	Yes, diagnosed by the vet	Yes, self-diagnosis	No	I don't know
**Has your cat been diagnosed with a nervous system-related problem or illness?**	○	○	○	○

Please indicate the illness/problem(s) below:

☐ Epilepsia

☐ Cerebellum degeneration (abiotrophy, ataxia etc.)

☐ Cerebellar hypoplasia (Wobbling kitten syndrome jne.)

☐ Central nervous system developmental problem

☐ Peripheral nervous system developmental problem

☐ Neuropathic pain

☐ Nystagmus

☐ PNS neural malfunction (polyneuropathy)

☐ Other nervous system related illness/problem (ATTENTION! Please clarify in the text box below!)

Please provide more information regarding the illness/problem (when it was diagnosed, medication, age of onset, symptoms etc)

19. Genital diseases

	Yes, diagnosed by the vet	Yes, self-diagnosis	No	I don't know
**Has your cat been diagnosed with a genital organ-related problem or illness?**	○	○	○	○

Please indicate the illness/problem(s) below

Female

- False/phantom pregnancy
- Uterine infection
- Pyometra
- Miscarriage
- Birthing complications
- Ceasarean section
- Stillborn kittens
- Infertility
- Loss of lipido
- Never in heat
- Continuously in heat
- Vaginal infection
- Mammary gland infection
- Ovarian cyst
- Female other illness/problem (ATTENTION! Please clarify in the text box below)

	Yes	No	I don't know
**Does the dam defend her kittens?**			

Male

- Undescended testis (How many? Please clarify in the text box below)
- Testis infection
- Lack of interesting/inability to mate. Please describe in the text box below!
- Male's other illness/problem (ATTENTION! Please clarify in the text box below)

	Never	Rarely	Sometimes	Often
**How often does you male cat mark?**				

Please provide more information regarding the illness/problem (when it was diagnosed, medication, age of onset, symptoms etc)

20. Endocrine and metabolic diseases

	Yes, diagnosed by the vet	Yes, self-diagnosis	No	I don't know
**Has your cat been diagnosed with a endocrinological (hormonal) or metabolism-related problem or illness?**				

Please indicate the illness/problem(s) below

- ☐ Food allergy
- ☐ Amyloidosis (liver)
- ☐ Amyloidosis (kidney)
- ☐ Diabetes Mellitus 1
- ☐ Diabetes Mellitus 2
- ☐ Diabetes Insipidus
- ☐ Hypokalemia
- ☐ Cushing's disease (hyperadrenocorticism)
- ☐ Hyperthyroidism
- ☐ Hypothyroidism
- ☐ Pyruvate kinase deficiency (PK)
- ☐ Hyperadrenocorticism
- ☐ Hypoadrenocorticism
- ☐ Lysosomal storage disorders (Mucopolysaccaridosis I/IV/VI/VII, Mucolipidosis II, Alpha Mannosidosis & fucosidosis, Niemann-Pick disease)
- ☐ Lipidosis
- ☐ GM1 (gangliosidosis 1)
- ☐ GM2 (gangliosidosis 2)
- ☐ GSD-IV (glycogen storage disorder)
- ☐ Addison's disease & hypoadrenocortism
- ☐ Other illness/disease (ATTENTION! Please clarifty in the text box below)

Please provide more information regarding the illness/problem (when it was diagnosed, medication, age of onset, symptoms etc)

21. Autoimmune diseases

	Yes, diagnosed by the vet	Yes, self-diagnosis	No	I don't know
**Has your cat been diagnosed with a autoimmune-related problem or illness?**	○	○	○	○

Please indicate the illness/problem(s) below

- ☐ AIHA/IMHA (autoimmune hemolytic anemia)
- ☐ IMP (Immune-mediated thrombocytopenia)
- ☐ ITP (Idiopathic thrombocytopenia purpura)

☐ Other illness/problem (ATTENTION! Please clarify in the text box below)

Please provide more information regarding the illness/problem (when it was diagnosed, medication, age of onset, symptoms etc)

22. Tumors (including benign tumors and cancers)

	Yes, diagnosed by the doctor	Yes, own diagnosis	No	I don't know
**Has your cat been diagnosed with cancer or has tumors?**	○	○	○	○

Please indicate the illness/problem(s) below

☐ Mammary tumor

☐ Vaccine-associated sarcoma

☐ Liver tumor

☐ Oral cavity tumor

☐ Bone tumor

☐ Digestive tract tumor

☐ Testis tumor

☐ Skin tumor

☐ Other tumor (Attention! Please specify below in the textbox).

Please provide more information regarding the tumor/cancer (when it was diagnosed, medication, age of onset, symptoms etc)

23. Parasites and protozoans

	Yes, diagnosed by the vet	Yes, self-diagnosis	No	I don't know
**Has your cat been diagnosed with a parasite or fungal-related problem or illness?**	○	○	○	○

Please indicate the illness/problem(s) below

☐ External parasites (ex. ear mites, lice, fleas, demodex)

☐ Internal parasites (ex. tapeworm, roundworm, echinococcosis)

☐ Protozoans (ex. giardia, toxoplasma gondii, cryptosporidium)

Please provide more information regarding the parasite/protozoan infection (which parasite/protozoan,age of onset, symptoms, where it was diagnosed, care)

How often do you give your cat worming medication?

24. Diseases not mentioned in the previous categories

	Yes, diagnosed by the doctor	Yes, self-diagnosis	No	I don't know
**Has your cat been diagnosed with a problem or illness mentioned below?**	○	○	○	○

Please indicate the illness/problem(s) below:

- ☐ An allergy not already mentioned
- ☐ Disease/symptoms caused by foreign bodies
- ☐ FELV (Feline leukemia virus)
- ☐ FIP (Feline Infectious Peritonitis, FECV)
- ☐ FIV (Feline Immunodeficiency Virus)
- ☐ Feline upper respiratory infection (Feline Herpesvirus and Feline Calicivirus)
- ☐ FCV (Feline Calicivirus)
- ☐ FHV (Feline herpesvirus (the cause of feline viral rhinotracheitis))
- ☐ Feline panleukopenia (feline infectious/parvoviral enteritis)
- ☐ Another feline viral disease not mentioned
- ☐ Poisoning

Please provide more information regarding the illness/problem (when it was diagnosed, medication, age of onset, symptoms etc)

	Yes	No	I don't know	If so, to what?
**Does your cat show oversensitivity to anesthesia or sedation medications?**	○	○	○	
**Does your cat show oversensitivity to other medications?**	○	○	○	

Does your cat have an illness/problem(s) that were NOT mentioned in this questionnaire? If so, please clarify in the text box below the illness, age of onset, symptoms, etc

Did you remember to mention your cat's illness/problem(s) that were NOT mentioned in this questionnaire?

25. Vaccinations

	Yes	No	I don't know	If so, to which vaccine and what are the symptoms?
**Is your cat overly sensitive to vaccinations?**				
**Has your cat gone into anaphylactic shock after a vaccination?**				

Vaccinations given to your cat. Check those vaccinations given:

	Yes	No	I don't know
**FFeline Herpesvirus and Feline Calicivirus vaccine in the past year**			
**Feline panleukopenia vaccine in the past three years**			
**Feline chlamydia vaccine in the past year**			
**Feline leukemia vaccine in the past year**			
**Rabies vaccine in the past three years**			

Additional information regarding your cats vaccinations

Thank you for filling out this valuable questionnaire!

☐ If you don't want your cat's information to be given to the breed organization, select here (Administrator will be different than that of this questionnaire).

☐ I am interested in donating a blood sample from my cat to your DNA-bank. I want more information.

Questionnaire-related feedback and comments

Thank you for your time!
